# Editorial: Endocrinology in Cancer and Aging

**DOI:** 10.3389/fendo.2021.722929

**Published:** 2021-07-15

**Authors:** Ralf Jockers, Jianfeng Liu

**Affiliations:** ^1^ Université de Paris, Institut Cochin, INSERM, CNRS, Paris, France; ^2^ Cellular Signaling Laboratory, International Research Center for Sensory Biology and Technology of MOST, Key Laboratory of Molecular Biophysics of Ministry of Education, School of Life Science and Technology, Huazhong University of Science and Technology, Wuhan, China

**Keywords:** diabetes, obesity, dementia, pancreas, breast cancer

The Research Topic on “Endocrinology in Cancer and Aging” associated to the 7th edition of the International Congress on « Innovative therapeutics for Cancer and Ageing diseases » held in Wuhan (China) in October, 2019 generated a lot of interest with currently (June 2020) more than 22,000 views and more than 4,700 downloads of the 15 articles of this Research Topic.

Two articles of the Research Topic are authoritative review articles written by international experts in their fields. Duvillié et al. report on the interplay between type 2 diabetes and pancreatic ductal adenocarcinoma and insulinoma. The authors expose the complex landscape of the bidirectional interaction between diabetes and cancer that includes also obesity as a risk factor for diabetes, the influence of aging, genetic risk factors and pharmacological treatments. The authors try to consolidate the multiple interactions on the cellular and molecular level. Strous et al. focus in their review on growth hormone receptor (GHR) signaling pathways playing important roles in growth, metabolism, cancer and aging. As a cytokine receptor, GHR is regulated by signaling effectors that promote (i.e. JAK/STAT) or inhibiting (i.e. SOCS) GHR signaling as well as by ubiquitination followed by receptor proteasomal degradation, endocytosis and lysosomal degradation. The significance of GHR signaling for cancer development is demonstrated by mutations in the SOCS2-GHR axis that increases lung cancer risk as pointed out by the authors.

Several original articles of this Research Topic are studying the relation between diabetes and cancer in humans. In a prospective cohort study of 37,993 cancer survivors, Tao et al. found that diabetes increases the risk of all-cause mortality among breast, prostate, and colorectal cancer survivors but not for pre- or post-diagnosis diabetes.

In a case-control study on approximately 300 women with endometrial or ovarian cancer and a similar number of matched controls Liu et al. report that women with a history of gestational diabetes mellitus (GDM) or with the delivery of “large for gestational age” infants show increased the risk of developing endometrial cancer but not ovarian cancer. In contrast, the increase in risk of endometrial cancer was not observed in women with a history of preeclampsia. The authors speculate that increased placental leptin expression observed in GDM but not in preeclampsia might explain this difference.

Two association studies focused on elderlies. The study of Li et al. shows an association between polymorphisms in the Apolipoprotein E (*APOE*) gene, an important phospholipid and cholesterol transporter, with less depressive symptoms and higher serum LDL in Chinese elderly patients with schizophrenia, and a negative correlation between depressive symptoms and LDL. The study of Roh et al. shows that high body weight variability, i.e. repeated loss and regain of weight, is associated with an increased risk of dementia in a cohort of 19,987 elderly Korean. These studies highlight the increasingly recognized impact of metabolic dysfunction on dementia and schizophrenia.

Other articles report new biomarkers and advances in therapeutic solutions. Huang et al. analyzed the exosomal proteins present in the urine of 16 patients with papillary thyroid carcinoma and follicular thyroid carcinoma. By comparing the profiles before and after operation they identified serum thyroglobulin as a new biomarker for thyroid cancer. In a collection of 183 familial pancreatic neuroendocrine tumors Cuthbertson et al. showed that Ga-68 PET/CT imaging, a method based on the detection of somatostatin receptors present in 70-90 percent of neuroendocrine tumors, more accurately stages and guides the treatment of these patients.

In a retrospective study with 50 patients Wang et al. conclude that early diagnosis of primary adrenal lymphoma depends on a combination of biochemical examination, imaging studies, and pathological biopsy, and that a combined chemotherapy is associated with a good prognosis. Similarly, 58 patients with primary thyroid diffuse large B cell lymphoma having received a combination chemotherapy with radiotherapy had a better prognosis as reported by Yi et al. in a retrospective study.

The Research Topic includes also two insightful case reports. One on a 47-year-old female patient with primary adenoid cystic carcinoma of the upper anterior mediastinum mimicking a thyroid tumor (Wu et al.) and another on a 67-year-old man with a history of follicular thyroid carcinoma and papillary thyroid microcarcinoma (Wang et al.).

One original article uses an animal model, the MMTV-PyVT transgenic mouse model of spontaneous mammary tumors, to pinpoint the upregulation of quinolinate phosphoribosyltransferase (QPRT), an enzyme participating in NAD+ homeostasis (Liu et al.). Complementary *in vitro* studies in breast cancer cells indicate that QPRT enhanced breast cancer invasiveness probably through purinergic signaling and myosin light chain phosphorylation.

The two last original articles focus on *in vitro* studies to better understand the molecular mechanisms of cancer development. In the first study Wang et al. wanted to understand the effect of 17β-estradiol (E2) in the treatment of pelvic organ prolapse (POP) by determining the proliferation, apoptosis, and protein expressions of fibroblasts. The authors show that E2 can inhibit the progress of POP by inhibiting the expression level of mitofusin-2, a key regulator of mitochondrial fusion and division (Wang et al.). In second *in vitro* study starts from Czogalla et al. with a histological observation. The authors analyzed the expression of the scaffolding protein β-arrestin 2 in 156 patients with ovarian cancer. They show that high β-arrestin 2 expression correlates with high-grade serous histology and the expression of the gonadotropin receptors FSHR and LHCGR, as well as the membrane estrogen receptor GPER and hCGβ (Czogalla et al.). These results were confirmed by *in vitro* experiments in several cell lines. The authors propose β-arrestin 2 as a prognostic factor and a promising target for new therapeutic approaches in advanced ovarian cancer.

In conclusion, this Research Topic spans the full range of articles from case reports to studies on large cohorts, from original to review articles, from animal to human studies and from *in vitro* to *in vivo* studies. Altogether this collection makes a significant contribution to our understanding of endocrinology in cancer and aging.

## Author Contributions

Both authors contributed to the writing. All authors contributed to the article and approved the submitted version.

## Conflict of Interest

The authors declare that the research was conducted in the absence of any commercial or financial relationships that could be construed as a potential conflict of interest.

